# [Retracted] Cdc42 expression in cervical cancer and its effects on cervical tumor invasion and migration

**DOI:** 10.3892/ijo.2025.5833

**Published:** 2025-12-05

**Authors:** Hongnan Ye, Youyi Zhang, Li Geng, Zijian Li

Int J Oncol 46: 757-763, 2015; DOI: 10.3892/ijo.2014.2748

Following the publication of this paper, it was drawn to the Editor's attention by a concerned reader that, for the transfection experiments shown in Fig. 3 on p. 760, two pairs of data panels showed a surprisingly high level of similarity, such that the data appeared to have been derived from the same original sources where the results from differently performed experiments were intended to have been shown. The authors responded to the reader's concern by providing replacement data for Fig. 3; however, upon performing an independent analysis of the data in this paper in the Editorial Office, it was also noted that western blot data were overlapping in Fig. 4, and for the cell invasion assay experiments shown in Fig. 5, Fig. 5A and B were also found to contain overlapping data, albeit the level of brightness of the images differed.

Owing to the large number of duplications of data, and other potential anomalies, that were identified in this paper, the Editor of *International Journal of Oncology* has decided that it should be retracted from the Journal on account of a lack of confidence in the presented data. The authors were asked for an explanation to account for these additional concerns, but the Editorial Office did not receive a reply. The Editor apologizes to the readership for any inconvenience caused.

